# Patient safety in primary health care and polypharmacy: cross-sectional survey among patients with chronic diseases*

**DOI:** 10.1590/1518-8345.3123.3217

**Published:** 2019-12-05

**Authors:** Lorena Ulhôa Araújo, Delba Fonseca Santos, Emerson Cotta Bodevan, Hellen Lilliane da Cruz, Jacqueline de Souza, Neila Márcia Silva-Barcellos

**Affiliations:** 1 Universidade Federal dos Vales do Jequitinhonha e Mucuri, Departamento de Farmácia, Diamantina, Minas Gerais, Brazil.; 2 Universidade Federal dos Vales do Jequitinhonha e Mucuri, Departamento de Matemática e Estatística, Diamantina, Minas Gerais, Brazil.; 3 Universidade Federal de Ouro Preto, Escola de Farmácia, Ouro Preto, Minas Gerais, Brazil.

**Keywords:** Pharmacoepidemiology, Polypharmacy, Potentially Inappropriate Medication List, Primary Health Care, Patient Safety, Chronic Disease, Farmacoepidemiologia, Polifarmácia, Lista de Medicamentos Potencialmente Inapropriados, Atenção Primária à Saúde, Segurança do Paciente, Doença Crônica, Farmacoepidemiología, Polifarmacia, Lista de Medicamentos Potencialmente Inapropiados, Atención Primaria de Salud, Seguridad del Paciente, Enfermedad Crónica

## Abstract

**Objective:**

to characterize and determine the polypharmacy prevalence in patients with chronic diseases and to identify the factors associated, in order to improvement of pharmaceutical care focused on patient safety.

**Methods:**

cross-sectional study included 558 patients, covered by primary health care, using a household and structured questionnaire. We analyzed the data on polypharmacy and its clinical and socioeconomic factors. Poisson regression analysis with robust variance was applied, with results expressed in prevalence ratio.

**Results:**

the results showed that polypharmacy (consumption of four or more drugs) was of 37.6%. The prevalence ratio analyses identified independent variables associated with polypharmacy: age (3.05), economic strata (0.33), way of medication acquisition through a combination of out-of-pocket and Brazilian public health system (1.44), diabetes and hypertension (2.11), comorbidities (coronary artery disease 2.26) and hospital admission (1.73). In the analyses, inappropriate medication use of the 278 patients (≥ 65 years) was associated with polypharmacy (prevalence ratio 4.04).

**Conclusion:**

polypharmacy study becomes an opportunity to guide the strategies for the patient safety to promote the medication without harm in chronic diseases.

## Abstract (pt)

**Objetivo:**

caracterizar e determinar a prevalência de polimedicação em pacientes com doenças crônicas e identificar os fatores associados, buscando a melhoria da assistência farmacêutica com foco na segurança do paciente.

**Métodos:**

estudo transversal que incluiu 558 pacientes, cobertos pela Atenção Primária à Saúde, utilizando questionário estruturado aplicado em domícilio. Analisamos os dados sobre polimedicação, assim como seus fatores clínicos e socioeconômicos. A análise de regressão de Poisson com variância robusta foi aplicada, com resultados expressos em razão de prevalência.

**Resultados:**

os resultados mostraram que a prevalência de polimedicação (consumo rotineiro ou simultâneo de quatro ou mais medicamentos) foi de 37,6%. As análises de razão de prevalência identificaram as seguintes variáveis independentes associadas à polimedicação: idade (3,05), estrato econômico (0,33), forma de aquisição de medicamentos, que combinou compra do próprio bolso e acesso a partir do sistema de saúde pública brasileiro (1,44), diabetes e hipertensão (2,11), comorbidades (doença arterial coronariana, 2,26) e internação hospitalar (1,73). As análises mostraram que o uso inadequado de medicamentos por parte de 278 pacientes (≥ 65 anos) estava associado à polimedicação (razão de prevalência 4,04).

**Conclusão:**

o estudo da polimedicação é uma oportunidade para reorientar as estratégias de segurança do paciente, promovendo o uso de medicação adequada sem causar iatrogenia.

## Abstract (es)

**Objetivo:**

caracterizar y determinar la prevalencia de la polimedicación en pacientes con enfermedades crónicas e identificar los factores asociados, para mejorar la atención farmacéutica con un enfoque en la seguridad del paciente.

**Métodos:**

estudio transversal en el que participaron 558 pacientes, atendidos en la Atención Primaria de Salud, y que utilizó un cuestionario estructurado aplicado en el hogar. Se analizaron los datos sobre polimedicación, así como sus factores clínicos y socioeconómicos. Se aplicó el análisis de regresión de Poisson con varianza robusta, con resultados expresados como razones de prevalencia.

**Resultados:**

los resultados revelaron que la prevalencia de la polimedicación (consumo rutinario o simultáneo de cuatro o más medicamentos) fue del 37,6%. Los análisis de la razón de prevalencia identificaron las siguientes variables independientes asociadas con la polimedicación: edad (3,05); estrato económico (0,33); medios para comprar medicamentos, que combinó la compra por el propio usuario y su acceso desde el sistema de salud público brasileño (1,44); diabetes e hipertensión (2,11); comorbilidades (enfermedad coronaria, 2,26) y hospitalización (1,73). Los análisis revelaron que el uso inadecuado de medicamentos por 278 pacientes (≥ 65 años) estuvo asociado con la polimedicación (razón de prevalencia 4,04).

**Conclusión:**

el estudio sobre la polimedicación es una oportunidad para reorientar las estrategias de seguridad del paciente, promoviendo un uso adecuado de la medicación sin causarles grandes daños.

## Introduction

The World Health Organization (WHO), in 2017, has highlighted the polypharmacy as one of the key focus areas of its Third Global Patient Safety Challenge, Medication Without Harm. The term polypharmacy, which is the routine use of four or more medications, represents a main patient safety issue^(^ 1 ^)^. In this context, it is necesary a team-based care approach patient centered, such as strategy for clinical decision making, collaboration, adherence to prescribed regimen and monitoring. Team-based care includes the patient, the patient’s primary care provider, and other professionals, such as cardiologists, nurses and pharmacists^(^ 2 ^)^. In primary health care (PHC) the risks to patients are much more related to the lack of monitoring during prolonged periods and the difficulties of access to health care. In the hospital setting, the patient safety is the comum practice, currently evolving towards primary care^(^ 3 ^)^.

Aditionally, the use of multiple drugs has been associated with an increased risk for potentially inappropriate medication (PIM). Studies have observed a relationship between polypharmacy and various factors including age and health status^(^ 4 ^)^.

Recognition of polypharmacy is the first step towards irracional use prevention^(^ 5 ^)^. However, it is necessary to know the possibilities of evaluating drug-related problems and increasing their safe and effective use. In this context, a systematic review on the use of PIM showed the impact of the intervention of pharmacists and the sensibilzation of physicians regarding the innappropriate prescription, contributing to the management of PHC to the patient^(^ 6 ^)^.

In Brazil, the pharmaceutical profession underwent important transformations, with a change of roles and focus on the provision of clinical services^(^ 7 ^)^. Regulatory advances defined the pharmacy as a health entity dedicated to providing a pharmaceutical service to promote better health care for patients^(^ 8 ^)^. In this reality, it is important to note that these services have gained momentum by implementing the models focused on patient-centered. In parallel, in 2017, new regulations were implemented in pharmaceutical education, covering three axes: Health Care, Technology and Innovation in Health and Health Management^(^ 9 ^)^.

Important aspects of these implementations were reached in the field of pharmaceutical policies in PHC in the Brazilian Unified Health System (SUS)^(^ 10 ^)^. However, it imposes, in particular on the pharmacist, the need to advance in the qualification of the care offered to drugs users. In addition, the Ministry of Health establishes the National Patient Safety Program, and in 2017, within this Program established Safety Protocol in the prescription, use and administration of drugs^(^ 11 ^)^.

The objective of this study was to characterize and determine the prevalence of polypharmacy in patients with chronic diseases, and to identify the factors associated with it, in order to provide subsidies for the improvement of pharmaceutical care focused on patient safety in the PHC of the SUS.

## Method

A cross-sectional population-based study was performed in the urban area of Diamantina, Minas Gerais (MG), Brazil, with an estimated population of 45,880 inhabitants in 2010. Regarding the Human Development Index of the municipality studied, it was 0.716, reflecting indicators of education, housing, health, work, income, and vulnerability^(^ 12 ^)^. The population is covered by ten units of PHC in the SUS, which included almost 30.805 people, of which 4.922 were users with diabetes and/or hypertension.

All hypertensive and diabetic users, aged 18 years or over, covered by Diamantina PHC were eligible for the study. We excluded vulnerable subjects such as pregnant women and mental patients. In addition, patients coming from hospitals and who were hospitalized during the study were also excluded, because they could overestimate of polypharmacy. For the analysis, the sample size was determined by estimation of population prevalence of formula (finite population), with a precision of 5% and a 95% level of confidence.

The data were collected from January and July 2015. Face-to-face interviews were conducted with users, using a household and structured questionnaire, applied by a properly trained technical team.

The dependent variables were the presence of polypharmacy (consumption of four or more drugs prescriptions) and PIM use. The independent variables were: sex (female; male), age in years according to five categories (18-30; 31-45; 46-60; 61-75; ≥76), race/skin color (white; not white), marital status (married or cohabiting; single), number of household residents (1-3; 4-5; ≥6) , economic strata, classified according to the Brazilian Association of Market Research Companies (ABEP) (social classes A; B; C; D-E; A is the wealthiest class, and E is the poorest class)^(^ 13 ^)^, education in years of schooling (0-2; 3-5; 6-8; 9-11; ≥12), drug orientation, way of medication acquisition (out-of-pocket; out-of-pocket and SUS; SUS; copayment system), self-care with medication, chronic disease (hypertension; diabetes; diabetes and hypertension), comorbidities (stroke; arrhythmia; hypercholesterolemia; depression; coronary artery disease; obesity), medical assistance (health insurance; SUS), time of last medical consutation in months (<3; ≥3), hospital admission, alcohol consumption, smoking, physical activity, reduction in the consumption of salt and sugar, self-reported health (good; poor), usual activities and pain/discomfort.

All the drugs were classified into pharmacological groups using the WHO Anatomical Therapeutic Chemical Classification System (ATC)^(^ 14 ^)^. Each participant’s medication list, people aged 65 years or over, was evaluated for use of PIM by using the Beers’ Criteria^(^ 15 ^)^.

All data were analyzes with the assistance of the R software (version 3.3.0). Bivariate analyses were performed after individuals were stratified into two groups according to the presence of polypharmacy, by Pearson *Chi*-Square and Fisher Exact Tests. Multivariate analyses were performed by Poisson regression analysis, with robust variance, and were done to assess the association between independent variables and polypharmacy, and between independent variables and use of PIM. Variables with *p*<0.2 were maintained in the model to control confounding factors. The significance of the results is represented as a *p*<0.05.

The project was approved by the Federal University Human Research Ethics Committee (protocol number 060/12). Data collection began only after the subject accepted to participate in the study by signing the Informed Consent Form, without conflict of interest.

## Results

In the ten primary care units, 558 patients aged 18 years or older participated in the observational study period.

The principal characteristics (statistically significant with *p* < 0.05) of the participants are illustrated in Tables 1 and 2. Of these, women represented 68.3%, and individuals from 61 years or older constituted 60.9% (mean age = 63.4 ± 13.0 years old). These participants, 210 users (37.6%) presented polypharmacy, i.e. 4 or more drugs, with a higher proportion of women (79.5%).

**Table 1:** Socioeconomic characteristics of the study population. Diamantina, MG, Brazil, 2015 (n*=558)

Variable	%	Polypharmacy		*p*-value
		Yes (n*=210)	No (n*=348)	
Sex				< 0,001^†^
Female	68,3	79,5	61,5	
Male	31,7	20,5	38,5	
Age (years)				< 0,001^‡^
18-30	1.3	0,0	2,0	
31-45	6,6	2,4	9,2	
46-60	31,2	26,2	34,2	
61-75	42,6	49,0	38,8	
≥ 76	18,3	22,4	15,8	
Marital status				< 0,001^†^
Married or cohabiting	58,1	48,1	64,1	
Single^§^	41,9	51,9	35,9	
Economic strata^‖^				< 0,001^‡^
A	0,2	0,5	0,0	
B	10,8	5,7	13,8	
C	50,3	44,8	53,7	
D e E	38,7	49,0	32,5	
Education (years)				0,0119^†^
0-2	31,4	36,2	28,4	
3-5	32,9	37,2	30,5	
6-8	18,3	15,2	20,1	
9-11	9,7	6,2	11,8	
≥ 12	7,7	5,2	9,2	

* n = number of patients;

† Pearson Chi-Square Test;

‡ Fisher Exact Test; Statistically significant p-value < 0.05;

§ Includes unmarried, separated, divorced and widowed;

‖ Brazil criterion of economic classification

**Table 2:** Clinical and biological characteristics of the study population. Diamantina, MG, Brazil, 2015 (n*=558)

Variable	%	Polypharmacy		*p*-value
		Yes (n*=210)	No (n*=348)	
Medication				
Way of acquisition				< 0,001^†^
Out-of-pocket	23,1	21,4	24,1	
Out-of-pocket and SUS^‡^	10,0	21,9	2,9	
SUS^‡^	63,8	53,8	69,8	
Copayment system^§^	3,0	2,9	3,2	
Self-care				< 0,001^†^
Yes	90,7	83,3	95,1	
No	9,3	16,7	4,9	
Diseases				< 0,001^†^
Hypertension	52,3	34,8	62,9	
Diabetes	15,1	9,0	18,7	
Diabetes and hypertension	32,6	56,2	18,4	
Comorbidities				
Stroke				0,0049^†^
Yes	6,1	10,0	3,7	
No	93,9	90,0	96,3	
Hypercholesterolemia				0,0026^†^
Yes	4,1	7,6	2,0	
No	95,9	92,4	98,0	
Depression				0,0051^†^
Yes	5,7	9,5	3,4	
No	94,3	90,5	96,6	
Coronary artery disease				0,0240^‖^
Yes	2,0	3,8	0,9	
No	98,0	96,2	99,1	
Acess to medical assistance				
Medical consultation (months)				0,0338^†^
<3	59,3	65,2	55,7	
≥3	40,7	34,8	44,3	
Hospital admission				< 0,001^†^
Yes	15,2	26,7	8,3	
No	84,8	73,3	91,7	
Lifestyle and Health state				
Alcohol consumption				< 0,001^†^
Yes	24,4	15,7	29,6	
No	75,6	84,3	70,4	
Self-reported health				0,0130^†^
Good	85,5	80,5	88,5	
Poor	14,5	19,5	11,5	
Pain / discomfort				< 0,001^†^
Yes	49,1	58,6	43,4	
No	50,9	41,4	56,6	

* n = number of patients;

† Pearson Chi-Square Test; Statistically significant p-value < 0.05;

‡ SUS = Brazilian Unified Health System;

§ Copayment system - Brazilian Popular Pharmacy;

‖ Fisher Exact Test

A significant association was observed between polypharmacy and the following variables (Tables 1 and 2): sex, age, marital status, economic strata, education, way of medication acquisition, self-care with medication, chronic diseases, comorbidities (stroke, hypercholesterolemia, depression and coronary artery disease), medical consultation, hospital admission, alcohol consumption, self-reported health and pain/discomfort.

There were no significant association between polypharmacy and the following variables: race/skin color (*p*=0.8158), number of household residents (*p*=0.3399), drug orientation (*p*=0.1765), arrhythmia (*p*=0.0543); obesity (*p*=0.3188), medical assistance (*p*=0.5065), smoking (*p*=0.0648), physical activity (*p*=0.8138) and reduction in the consumption of salt and sugar (*p*=0.6469).

After adjustment for the covariables, the statistically significant variables (*p*<0.05) are presented in Table 3.

**Table 3:** Prevalence, prevalence ratio and analysis of factors associated with polypharmacy by Poisson regression model with robust variance. Diamantina, MG, Brazil, 2015 (n*= 210)

Variable	% of polypharmacy	PR^†^	95%CI^‡^	*p*-value
Age (years)				
18-45	11,4	1,00	-	-
46-60	31,6	2,56	1,25-5,21	< 0,01
61-75	43,3	3,05	1,49-6,24	< 0,01
≥76	46,1	2,73	1,29-5,78	< 0,01
Economic strata^§^				
A	100,0	1,00	-	-
B	20,0	0,17	0,09-0,31	< 0,001
C	33,5	0,26	0,14-0,49	< 0,001
D and E	47,7	0,33	0,17-0,65	< 0,01
Medication				
Way of acquisition				
Out-of-pocket	34,9	1,00	-	-
Out-of-pocket and SUS^‖^	82,1	1,44	1,07-1,94	0,01
Diseases				
Hypertension	24,8	1,00	-	-
Diabetes and hypertension	64,8	2,11	1,67-2,66	< 0,001
Comorbidities				
Stroke				
Yes	61,8	1,64	1,22-2,21	< 0,001
No	36,1	1,00	-	-
Arrhythmia				
Yes	58,3	1,56	1,07-2,27	0,02
No	36,7	1,00	-	-
Hypercholesterolemia				
Yes	69,6	1,43	1,02-2,01	0,03
No	36,3	1,00	-	-
Coronary artery disease				
Yes	72,7	2,26	1,37-3,72	< 0,01
No	36,9	1,00	-	-
Acess to medical assistance				
Hospital admission				
Yes	65,9	1,73	1,41-2,11	< 0,001
No	32,6	1,00	-	-

* n = number of patients;

† PR = Prevalence ratio;

‡ Confidence interval; Poisson regression, statistically significant p-value < 0.05; Only independent variables with p-value < 0.20 in the univariate analysis were included in the multivariate model;

§ Brazil criterion of economic classification;

‖ SUS = Brazilian Unified Health System

The patients with polypharmacy were associated with age, economic strata, way of medication acquisition through a combination of out-of-pocket and SUS, chronic diseases (diabetes and hypertension), comorbidities (stroke, arrhythmia, hypercholesterolemia, coronary artery disease) and hospital admission (Table 3).

The polypharmacy was no association with following variables: sex, marital status, education, drug orientation, way of medication acquisition by SUS and by copayment system (Brazilian Popular Pharmacy), self-care with medication, diabetes chronic disease, depression comorbidity, medical consultation, alcohol consumption, smoking, self-reported health, usual activities and pain/discomfort.

There were 114 patients (41.0%), aged over 65 years, treated with PIM during the time of this study, among which 51 patients (44.7%) using a single, 51 (44.7%) using two, while 12 (10.6%) were using 3 to 4. In total, the elderly patients used 23 different PIM.

The PIM most commonly used, according ATC classification, have been those which act on the cardiovascular system (38.6%): spironolactone (9.2%), nifedipine (7.4%), digoxin (7.4%), methyldopa (6.7%) and amiodarone (3.7%). The second most used group was the drugs that act on the blood and blood forming organs (38.0%): aspirin (36.2%). Drugs that act on the nervous system accounted for 19.6% of potentially inappropriate medications used, and of these, the most used was clonazepam (4.3%), followed by paroxetine (3.1%), amitriptyline (3.1%) and diazepam (2.4%).

Table 4 shows that elderly patients who were using more than 4 medications were 4 times more likely to receive a PIM than those who were using three or fewer medications, prevalence ratio (PR) 4.04, 95% confidence interval (CI) 2.76-5.92, *p*<0.001. Among elderly patients with polypharmacy, 77.2% used PIM. There was no association between age, chronic disease and comorbidities in receiving a PIM.

**Table 4:** Principal characteristics of elderly patients taking inappropriate medications versus those not taking inappropriate medications. Diamantina, MG, Brazil, 2015 (n* = 278)

Variables	Medication use		PR^†^	95%CI^‡^	*p*-value
	Inappropriate (n*= 114)	Appropriate (n*= 164)			
Age (years)					
65-74	68	100	1,00	-	-
75-84	34	49	1,03	0,80-1,34	0,80
≥85	12	15	1,04	0,68-1,57	0,87
Disease					
Hypertension	53	95	1,00	-	-
Diabetes	12	24	0,87	0,61-1,22	0,42
Diabetes and hypertension	49	45	0,91	0,70-1,18	0,49
Comorbidities					
Yes	34	25	1,13	0,86-1,47	0,38
No	80	139	1,00	-	-
Polypharmacy					
Yes	88	39	4,04	2,76-5,92	< 0,001
No	26	125	1,00	-	-

* n = number of patients;

† PR = Prevalence ratio;

‡ Confidence interval; Poisson regression, statistically significant p-value < 0.05

## Discussion

The main results of this study demonstrate of the prevalence of drug use, polypharmacy and PIM use in the PHC. The predominance was of drugs for the cardiovascular system, which may develop risks for adverse events due to polypharmacy^(^ 16 ^)^. In this situation, the drugs rational use by this group requires doses that meet their needs according to the international guidelines of medication without harm.

According to the Guideline for Prevention, Detection, Evaluation and Management of Adult Hypertension, PHC to the patient by pharmacists and others professionals should be share^(^ 2 ^)^. The importance of pharmaceutical services is recognized, that allowed patients with chronic conditions and polymedicated to have access to the pharmacotherapeutic follow up^(^ 7 ^)^. In this context, monitoring polypharmacy is a primary care practice that can contribute to reduce of side effects, as well as the risks of interactions, and improve adherence. This is important, because the polypharmacy has increased with greater life expectancy and as older people live with several chronic diseases^(^ 1 ^)^.

Referring to pharmaceutical care, the activities of clinical nature performed by pharmacists in Brazil are still incipient^(^ 17 ^)^. Futhermore, there are deficiencies in the workforce composition, which may affect the quality of the drug use and its results on health^(^ 18 ^)^. However, the pharmacists have expanded their roles and responsibilities in PHC through regulatory advancements.

During this study, diabetic and hypertensive users of PHC were prescribed with 3.3 drugs on average. Furthermore 37.6% of this population was exposed to polypharmacy. Several previous international studies have also reported the polypharmacy prevalence^(^ 19 ^-^ 20 ^)^. Among them, the results from a French study^(^ 20 ^)^ were very similar to our own. In Brazil, the study carried out in the city of Ribeirão Preto in the SUS showed that the polypharmacy prevalence was 47.9%^(^ 19 ^)^.

The data of this study displayed association between the polypharmacy prevalence and the socioeconomic variables. Indeed, polypharmacy increased with aging, growing from 43% of polymedicated people at 60 years old to 89.4% for people aged over 61 years old. Previous population-based studies also found an association between polypharmacy and older age^(^ 19 ^-^ 20 ^)^. In addition, the economic status of the study population was also associated. Thus, in order to implement clinical pharmacists practice is essential the knowledge of the socio-economic characteristics of patients for reduce barriers to counseling in the therapeutic orientation.

Among the respondents who reported using drugs, 31.7% had all the drugs they need through the SUS. This result was consistent with several studies that have shown the low acquisition of medications by SUS^(^ 21 ^-^ 23 ^)^, especially for the treatment of hypertension and diabetes^(^ 24 ^)^. In addition, Diamantina is a microregion located in the Jequitinhonha Valley, with low population density and few studies on access and use of drugs. So, it is necessary to emphasize that the results corroborate with the findings of another study^(^ 25 ^)^, in which the highest proportions of drug access were found in the more developed Brazilian regions, with a higher population density, observing and assessing the specificities of each region, especially in processes of regional planning of health actions.

Therefore, the access to drug by SUS combined with out-of-pocket (82.1%) is, consequently, associated with polypharmacy. In Brazil, there was high proportion of drug acess by purchase^(^ 26 ^)^, since 13% of patients reported not being able to buy something important to cover expenses with a health problems, and 41.8% of them pointed out the expense with drugs^(^ 27 ^)^.

In this context, this research carried out in a low-income region, such as Vale do Jequitinhonha, may support others works on access and the way of obtaining medicines. Whereas economic incentives and free provision by SUS of drugs may improve access and avoid high expenditures for drugs^(^ 28 ^)^.

Polypharmacy is a complex phenomenon, which helps distinguish a drug use for real health needs from not necessary use^(^ 29 ^)^. Therefore, it is necessary further research exploring their relationship with the SUS and health insurance. In Brazil, furthermore, economic crisis, health sector management and access to essential drug depend on how the drugs used were obtained^(^ 26 ^)^.

The expenses of the SUS with drugs grew, an important fact to be analyzed in a health system with polymedicated patients. Additionally, this issue is especially important for the country, since lower income families still proportionally commit more of their health care resources and drug spending has a significant share of these expenditures^(^ 27 ^)^.

This work explored the relationship between specific chronic diseases diagnosis and polypharmacy. Similar to previous Brazilian studies, high prevalence of chronic diseases, with hypertension and diabetes being the most common ones^(^ 19 ^,^ 30 ^)^. Multimorbidity is associated with a high number of prescribed medications^(^ 4 ^)^ and suggests relatively rapid changes in prescription patterns.

Similar to the current findings, hospital admission was associated with polypharmacy^(^ 31 ^)^. It is important to emphasize that pharmacotherapeutic success throughout from the performance of the pharmacist from the continuity of care in the health system. In this sense, a new paradigm for pharmaceutical education, defending the teaching focused on clinical skills regarding patient safety^(^ 32 ^)^. In Brazil, these changes in professional priorities are reflected barriers and facilitators that were grouped: health system, local healthcare network, pharmacists, health team, clinical pharmacy services implementation process and external factors^(^ 33 ^)^.

The progression of diabetes and hypertension can progress to the development of comorbidities, and as a consequence, polypharmacy. In this sense, elderly patients with diabetes represent a population with a high incidence of comorbidities and a reduced ability to tolerate medication adverse effects and drug-drug interactions^(^ 34 ^)^.

The pharmacotherapy process in polymedicated patients involves understanding the drug related problems, and is necessary the identification of these problems associated with PIM in elderly patients. As a consequence, Beers’ Criteria address several important aspects of PIM use in older people. This study demonstrates that 41.0% of diabetic and hypertensive elderly patients, who are covered by primary care, using at least one PIM, as determined by this Criteria^(^ 15 ^)^ and other studies have reported this use in Brazil^(^ 35 ^-^ 36 ^)^.

The PIM use may have unintended consequences of its effects, although there is evidence about the risks and harmful impact from its use. In the analysis of PIM grouped by ATC system, it was observed the use was the group of drugs that act on the cardiovascular system (38.6%). This is not surprising, since Brazilian population study has identified this class of drugs as the most used by the elderly^(^ 35 ^)^. Additionally, in another observational study conducted in Spain^(^ 37 ^)^, the most frequent drugs were prescribed for the cardiovascular (16.9%), gastrointestinal (15.5%) and musculoskeletal system (15.3%) and for medication-related central nervous system treatments (10.8%).

Spironolactone was the most used drug (9.2%) by the patients studied that acts on the cardiovascular system, and should be avoided with variation in the level of kidney function, since its administration is associated with increased potassium, according to Beers’ Criteria^(^ 15 ^)^. The use of nifedipine (immediate release) (7.4%) remains a concern because it is associated with hypotension and precipitating myocardial ischemia. This fact shows the importance of developing good practice guidelines for the prescription and use of these drugs in PHC, to ensure patient safety. In addition, digoxin (7.4%) used in atrial fibrillation or heart failure as a first-line agent should also be avoided because there are more-effective alternatives. The update of Beers’ Criteria confirms that methyldopa (6.7%) is not recommended as routine treatment for hypertension in the elderly due to high risk of adverse central nervous system effects, bradycardia and orthostatic hypotension. The use of aspirin (36.2%; prescribed at a dose of 100 mg) for primary prevention of cardiac events should be done with caution, especially for patients over 80 years of age, since lack of evidence of benefit versus risk. Due to the vulnerability of the interviewees’ age and the possibility of changing the medication from its original packaging, an unsafe dosage may be used. It is noted that this situation requires a continuous monitoring of pharmacotherapy in this age group. In this context, it is the pharmacist responsibility in the clinical practice to evaluate the treatment for vulnerable older people, as collaborative action in rational prescribing in PHC.

The findings showed that polypharmacy was common and was associated with PIM (95%CI 2.76-5.92; *p* <0.001). This result is consistent with findings in others studies, such as 95%CI 1.79-3.11; *p*<0.001^(^ 36 ^)^ and 95%CI 2.1-3.8; *p*<0.001^(^ 38 ^)^. The results of this study show the need for continuous pharmacotherapeutic monitoring. The integrated care is receiving more attention, and there is good evidence of the benefit of pharmacist involvement in drugs management^(^ 5 ^)^.

Beers’ Criteria is a clinical and public health tool to improve medication safety in older adults and to increase awareness of polypharmacy^(^ 15 ^)^. However, there may be cases in which the health care provider determines that a drug on the list is the only alternative. In this regard, there is the National List of Essential Medicines, used as a guideline for a rational prescription in PHC. However, to ensure the quality of primary care in the drug use it is necessary the use of the standardized list in order to contribute to the promotion of the quality of care^(^ 39 ^)^. Besides that, measurement of safety events, e.g. the polypharmacy and PIM use, in the primary care setting is essential, and the appropriate use of polypharmacy would be the indicator of drugs safety on potentially inappropriate prescription.

Therefore measures that qualify health, prescription, and dispensing services are needed to promote the rational use of drugs, in the PHC, through drugs optimisation^(^ 39 ^)^. The pharmacist role is undergoing transformation, and the graduates in Pharmacy should be able to engage in direct functions of clinical care. The data of this study also guide to relevant aspects of the scope of pharmaceutical education, focusing on the curriculum. Recommend the implementation of primary care protocols with newer practice models of care with main focus in polypharmacy control and reduction of PIM use.

One example of this newer practice model, according with the literature^(^ 40 ^)^, the deprescribing is the process of tapering or stopping drugs, aimed at minimizing polypharmacy and improving patient outcomes. Moreover, this process close collaboration with a team-based care approach patient centered.

The present study emphasizes the importance of periodic cross-sectional studies as generated data on drug use, thus evaluate health conditions and municipal health service performance. In this sense, the results found are in line with the study describes the development of the medication history of the medical records^(^ 41 ^)^. The pharmacoepidemiology is an important tool for the pharmacist to develop indicators for the rational use of drugs. In sequence they conduct the direction and the efficient control of clinical pharmacists practice in PHC. The polypharmacy is an indicator associated with socio-economic and clinical factors of the patient and can contribute in a significant way for the planning of clinical pharmacists practice within the holistic care of chronic patients in PHC.

This study may be limited by the regional differences in the polypharmacy prevalence that could be explained by regional age distributions, chronic diseases or by greater access to health care for patients living in Diamantina. In addition, given the cross-sectional nature of our study, it is impossible to determine the intensity and duration of polypharmacy outcome. Another aspect, fewer studies have examined whether lifestyle factors are associated with polypharmacy. For example, the variables of the subject’s lifestyle, such as alcohol consumption and smoking were obtained by self-report, and this method of data collection relies on all participants accurately and truthfully recalling information to prevent bias. However, the researchers recognize the need to develop detailed studies on lifestyle, health state and polypharmacy in primary health care. Longitudinal studies of counseling and lifestyle intervention are necessary for hypertensive and/or diabetic patients to obtain good clinical control and improve quality of life. Finally, as this is an exploratory study with limited statistical power, the domiciliary interviews may have biased the sample.

## Conclusion

Polypharmacy studies in PHC, including the general population, are scarce. The populations are aging and there are evidence about the relationship between increasing age and number of prescription drugs. In this aspect, increased morbidity and mortality and increased costs in health systems reinforce the need of health professionals in primary care must ensure the quality of pharmacotherapy.

Our study, in addition to confirming polypharmacy in hypertensive and diabetic patients and the association of inappropriate use of medications, was essential to reflect on the pharmacist’s role in the context of PHC. In this sense, the study of polypharmacy becomes a key element, an opportunity for pharmacists in partnership with primary care professionals to develop guidelines for the pharmacotherapy of chronic diseases, especially hypertension and diabetes. As a consequence, obtain better health outcomes based on patient safety in PHC.
